# Exogenous melatonin induces phenolic compounds production in *Linum album* cells by altering nitric oxide and salicylic acid

**DOI:** 10.1038/s41598-023-30954-9

**Published:** 2023-03-13

**Authors:** Sara Esmaeili, Mohsen Sharifi, Faezeh Ghanati, Bahram M. Soltani, Elaheh Samari, Mostafa Sagharyan

**Affiliations:** 1 Department of Plant Biology, Faculty of Biological Sciences, Tarbiat Modares University, Tehran, Iran; 2 Center of Excellence in Medicinal Plant Metabolites, Tarbiat Modares University, Tehran, Iran; 3 Department of Molecular Genetics, Faculty of Biological Sciences, Tarbiat Modares University, Tehran, Iran

**Keywords:** Biochemistry, Cell biology, Plant sciences

## Abstract

Melatonin is a pleiotropic molecule that can influence various aspects of plant performance. Recent studies have exhibited that it mediates plant defensive responses, probably through managing redox homeostasis. We tried to track the regulatory effects of melatonin on the antioxidant machinery of *Linum album* cell culture. To this, different concentrations of melatonin were applied, and the oxidative status of cells was investigated by measuring the levels of oxidative molecules and antioxidant agents. The results showed that H_2_O_2_ content did not change at the low melatonin levels, while it increased at the high concentrations. It can be correlated with the low melatonin dosages capacity to remove excessive amounts of H_2_O_2_, while the high melatonin dosages exhibit toxicity effects. In contrast, the NO enhancement occurred at 50 μM melatonin, proposing its role in triggering melatonin-induced defensive responses. The MDA results stated that NO led to oxidative stress in melatonin-treated cells at 50 μM melatonin. Antioxidant enzyme POD was activated by melatonin treatment, while SOD enzyme behaved reversely which can explain the changes in the H_2_O_2_ level. In addition, the analysis of the phenolics profile showed that the contents of phenolic acids, flavonoids, and lignans enhanced following an increase in PAL enzyme activity. The increased level of phenolic hormone SA can indicate that melatonin affects the defensive responses in *L*. *album* cells through a SA-dependent pathway. In general, it seems that melatonin, by modulating NO and SA levels, can induce the activity of antioxidant enzymes and the production of phenolics, especially lignans, in *L. album* cells.

## Introduction

Melatonin (*N-*acetyl-5-methoxytryptamine), an indole compound derived from serotonin, is a naturally pleiotropic molecule with different physiological functions in plants and animals^1,2^. In plants, it is assumed as a multifunctional regulatory molecule involved in numerous physiological processes such as root formation, seed germination, photosynthesis, circadian rhythms, leaf senescence delay, fruit ripening, and defensive responses^1,3–5^.

It has been stated that melatonin may increase in plants versus abiotic and biotic stresses, which is consistent with its key roles in activating defense reactions as a signaling and antioxidant molecule. In response to adverse conditions, plants undergo changes in cellular redox status as a result of reactive oxygen species (ROS) and reactive nitrogen species (RNS) production, through apoplastic and/or subcellular organelles reactions^6^. Oxidative stress can damage cellular components such as membranes, proteins, carbohydrates and nucleic acids^7,8^. Likewise, plants can cope with oxidative stress by evolving several efficient defensive reactions which exhibit crucial roles in preserving their survival^9,10^. Melatonin is known to be involved in maintaining cellular and intracellular redox homeostasis to regulate plant cell physiological function in response to stress conditions^11–13^. Recent studies have suggested that this molecules can directly enhance the cell antioxidant capacity and equips plants with different degrees of tolerance to biotic and abiotic stresses, such as drought, salinity, high light intensity, herbicides, and ultraviolet radiation^11,14,15^. Furthermore, since mitochondria and chloroplasts have been recently known the sources of melatonin production, it can act against various ROS in these compartments^16,17^. Previous studies have indicated that the application of melatonin induces the antioxidant enzymes activity such as superoxide dismutase (SOD), catalase (CAT), and peroxidase (POD) that is considered as a general mechanism to prevent the worst impacts of ROS on the cells^18^. This function of melatonin suggests its role as a signaling molecule in the regulation of plants’ defensive responses by influencing downstream signaling. Regarding this hypothesis, the interactions between melatonin and different signaling molecules such as mitogen-activated protein kinase (MAPK) cascades, Ca^2+^ influx, hydrogen peroxide (H_2_O_2_), nitric oxide (NO), and salicylic acid (SA) was previously observed^19^.

Besides, melatonin apparently acts as a phytohormone due to its structure, contributing to produce secondary metabolites more efficiently^20^. Recent studies have clearly shown that the melatonin application can impressively affect phenolics accumulation in the different plant species such as *Ocimum basilicum* var purpurascens^2^, and *Rosmarinus officinalis*^21^. It has been reported that melatonin can affect the contents of phenolic compounds through activation of enzymes involved in phenylpropanoid pathway^22^. Phenolic compounds (e.g., phenolic acids, flavonoids, and lignans) are characterized as natural products, linked to increasing the antioxidant capacity of the plants and protecting them from adverse conditions^23,24^. These compounds are derived from the phenylpropanoid pathway, where phenylalanine and tyrosine amino acids are converted into simple phenolic acids by the activities of Phenylalanine ammonia-lyase (PAL) and Tyrosine ammonia-lyase (TAL) enzymes, respectively^10^. A large body of studies is postulated that plants transiently increase the production of phenolic compounds in response to stresses^25,26^. Previous studies have pointed out that endogenous SA, a phenolic phytohormone, regulates many features of the plant defense machinery, including the biosynthesis of secondary metabolites^8^. Also, it has been suggested that melatonin and SA biosynthesis pathways compete for the chorismic acid precursor, which is provided by shikimic acid route^27^. According to the findings of Khan et al., 2015 and Wang et al., 2018, the protective roles of melatonin are probably related to its cross-talk with SA and other signaling molecules such as H_2_O_2_ and NO, which can improve the production of secondary metabolites and the activities of antioxidant enzymes to alleviate the oxidative stress^27–29^.

*Linum album* Kostchy Boiss., a native species of Iran, is a potent alternative source of phenolics, especially lignans, widely used as antiviral, anti-cancer, anti-tumor, and anti-inflammatory agents^30^. Lignans are an important subgroup of phenylpropanoids, leading to reinforce defense responses in this species^31^. However, till now, there are no scientific reports on the possible mechanisms by which melatonin regulates the defense responses of *L*. *album* cells*.* Therefore, in this study, the *L*. *album* cells were treated with different concentrations of melatonin (50, 100, and 150 μM) for 120 h. Subsequently, we analyzed the changes in the POD and SOD enzymes activities, H_2_O_2_, NO, and malondialdehyde (MDA) levels, and phenolic acids, flavonoids, and lignans quantification in the treated cells.

## Results

### Effect of melatonin on H_2_O_2_, NO, and MDA contents

**Figure 1:**
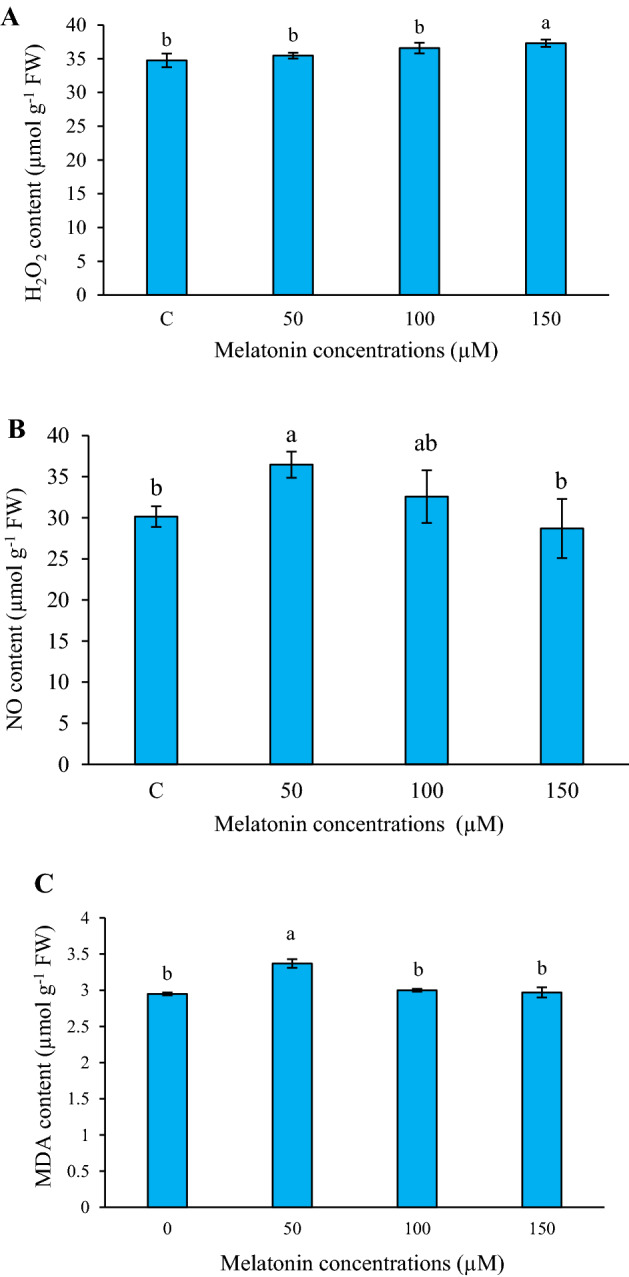
Effects of different concentrations of melatonin on H_2_O_2_ (**A**), NO (**B**), and MDA (**C**) contents in *L. album* cells. Means from 3 separate experiments ± SE. Means marked with the same letter were not significantly different according to Duncan's multiple range test (*p* value ≤ 0.05).

In the present study, changes in the oxidative status of *L*. *album* cells treated with different concentrations of melatonin were initially evaluated by measuring H_2_O_2_, NO, and MDA contents. Results of this experiment showed that under treatment condition, the content of H_2_O_2_ was increased at 150 µM melatonin (*p* ≤ 0.05), while low levels of melatonin did not significantly influence H_2_O_2_ content (Fig. 1A). In contrast, NO content was induced at low level of melatonin treatment, so that the highest amount of NO was observed in *L*. *album* cells under 50 µM melatonin compared to the control (Fig. 1B). Under melatonin treatment, the content of MDA was transiently enhanced, where the application of 50 µM melatonin induced it by 1.14-fold compared with the control (Fig. 1C). At low concentrations of melatonin, H_2_O_2_ content remained constant compared to the control. On the other hand, with a sudden increase in NO content at 50 µM melatonin, the formation of MDA was also significantly changed.

### Effect of melatonin on the activity of POD and SOD enzymes

**Figure 2:**
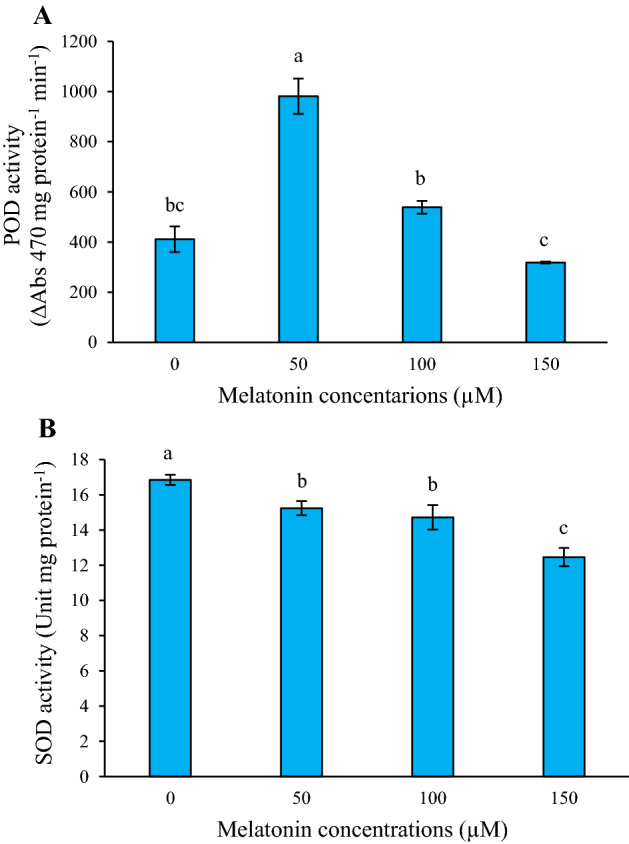
Effects of different concentrations of melatonin on the activities of POD (**A**), and SOD (**B**) enzymes in *L. album* cells. Means from 3 separate experiments ± SE. Means marked with the same letter were not significantly different according to Duncan's multiple range test (*p* value ≤ 0.05).

To better comprehend the effect of melatonin on enzymatic antioxidant responses, the activity of POD and SOD in the treated and untreated cells were tracked. As depicted in Fig. 2A, POD enzyme activity raised quickly after melatonin treatment compared to the control and reached its peak (approximately 2.38-fold higher than in the control cells) at 50 µM melatonin treatment. However, data showed that exposure to different concentrations of melatonin decreased the activity of SOD in the *L*. *album* cells, which was 1.35-fold less than the control sample at 150 µM melatonin (Fig. 2B).

### Effect of melatonin on PAL and TAL enzyme activities

**Figure 3:**
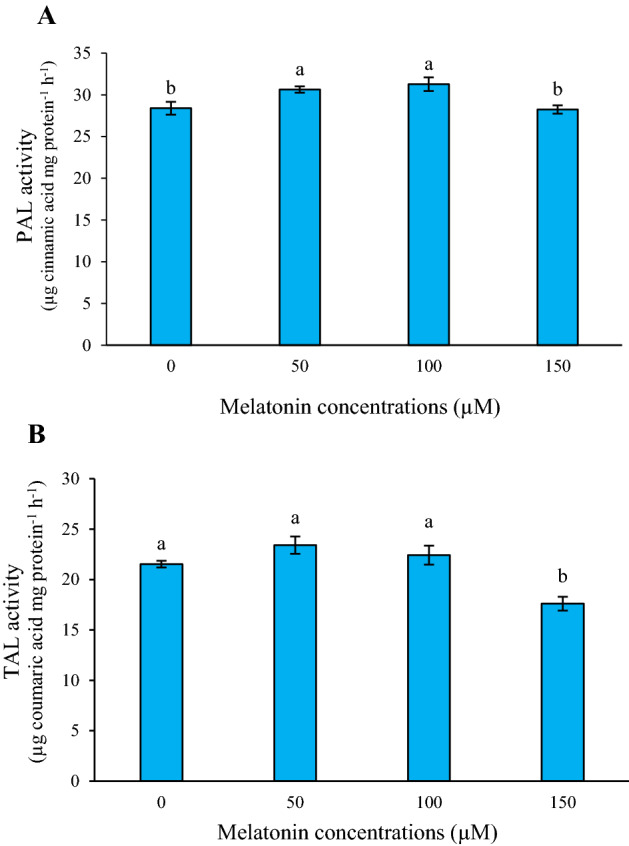
Effects of different concentrations of melatonin on the activities of PAL (**A**), and TAL (**B**) enzymes in *L. album* cells. Means from 3 separate experiments ± SE. Means marked with the same letter were not significantly different according to Duncan's multiple range test (*p* value ≤ 0.05).

In this study, the catalytic activity of PAL and TAL enzymes in *L*. *album* cells were determined. According to the results, PAL activity in the treated cells was induced at low concentrations of melatonin treatment, which reached its peak at 100 µM (Fig. 3A). In return, different concentrations of melatonin had no significant effect on the TAL enzyme activity relative to the control group (Fig. 3B). Moreover, the activity of this enzyme decreased at the high level of melatonin compared to the control sample.

### Effect of melatonin on phenolic acids and flavonoids accumulation

The pronounced effect of melatonin on the accumulation of individual phenolic acids and flavonoids were assessed through HPLC method. The results revealed that the content of caffeic acid was significantly impaired under treatment with different concentrations of melatonin in comparison with the control condition. However, the exact contrary trend was observed in the case of cinnamic acid. According to our data, the exogenous application of melatonin significantly increased cinnamic acid at 50 and 100 µM, which were by 1.50-fold and 1.60-fold compared to the control, respectively. On the other hand, the coumaric acid content in cells of *L*. *album* was raised by 50 µM melatonin treatment, but its level did not change significantly compared to the control. Moreover, ferulic acid and SA contents remarkably enhanced at 50 and 100 µM melatonin treatments, as well (Table 1).

**Table 1:** Effects of different concentrations of melatonin on the contents of phenolic acids and flavonoids in *L. album* cell culture.

Treatments (µM)	Phenolic acids					Flavonoids		
	Caffeic acid	Cinnamic acid	Coumaric acid	Ferulic acid	Salicylic acid	Catechin	Kaempferol	Resveratrol
0	1.402 ± 0.031^a^	6.170 ± 1.24^b^	1.478 ± 0.28^ab^	108 ± 13.54^b^	6.55 ± 2.94^b^	165 ± 18.5^a^	11.78 ± 0.34^b^	11.64 ± 0.63^b^
50	1.573 ± 0.69^c^	9.052 ± 1.26^a^	3.560 ± 1.44^a^	229 ± 51.66^a^	7.82 ± 0.94^a^	105 ± 4.15^b^	33.8 ± 5.5^a^	18.52 ± 0.69^a^
100	2.677 ± 0.20^b^	9.724 ± 4.42^a^	2.214 ± 0.105^b^	229 ± 24.91^a^	11.51 ± 1.81^a^	127 ± 2.56^b^	17.89 ± 0.30^b^	17.25 ± 0.10^a^
150	0.692 ± 0.14^c^	2.580 ± 0.24^c^	0.285 ± 0.35^c^	50 ± 12.49^c^	6.39 ± 0.84^b^	79 ± 19.53^c^	10.92 ± 1.18^b^	11.11 ± 0.76^b^

Means from 3 separate experiments ± SE. Means marked with the same letter were not significantly different according to Duncan's multiple range test (*p* value ≤ 0.05).

Meanwhile, the application of melatonin decreased catechin content, while significantly increased kaempferol level compared to the control after 120 h. Also, resveratrol changes were dependent on the melatonin concentrations, and accordingly, the amount of resveratrol reached its peak at 50 and 100 µM melatonin treatments (Table 1). Therefore, 50 and 100 µM melatonin treatment was an appropriate concentration to induce phenolic acids and flavonoids in *L*. *album* cells.

### Effect of melatonin on lignans production

**Figure 4:**
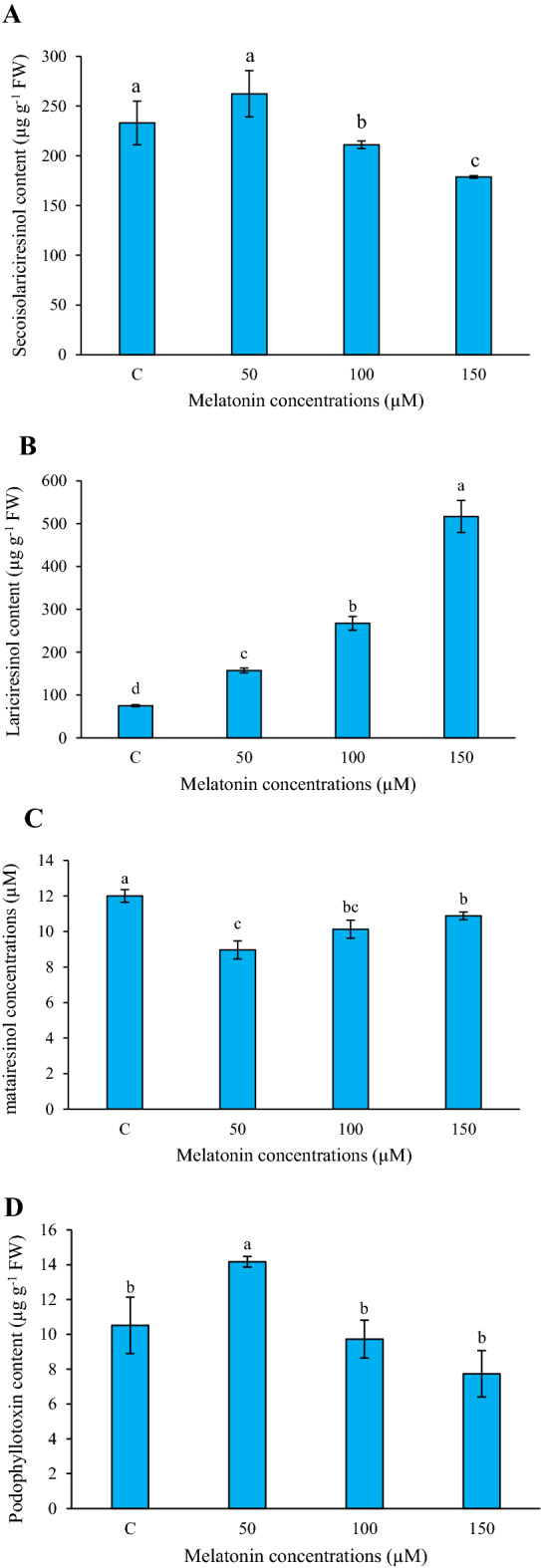
Effects of different concentrations of melatonin on secoisolariciresinol (**A**), lariciresinol (**B**), matairesinol (**C**), and podophyllotoxin contents in *L. album* cells. Means from 3 separate experiments ± SE. Means marked with the same letter were not significantly different according to Duncan's multiple range test (*p* value ≤ 0.05).

In the current work, the relative contents of four prominent lignans including secoisolariciresinol (SECO), lariciresinol (LARI), matairesinol (MATA), and podophyllotoxin (PTOX) were assayed through the HPLC system. As shown in Fig. 4A and C, the results emphasized that the SECO and MATA contents decreased at different concentrations of melatonin, so that their lowest levels were seen at 150 and 50 µM melatonin treatment, respectively. On the other hand, according to Fig. 4B, LARI content raised in all melatonin concentrations, while its level peaked at 150 µM melatonin treatment. Ultimately, PTOX enhancement occurred at 50 µM melatonin treatment, and its peak was about 1.53-fold in comparison to the control sample (Fig. 4D).

### Metabolic profiles of *L. album* cells in response to melatonin treatment

**Figure 5:**
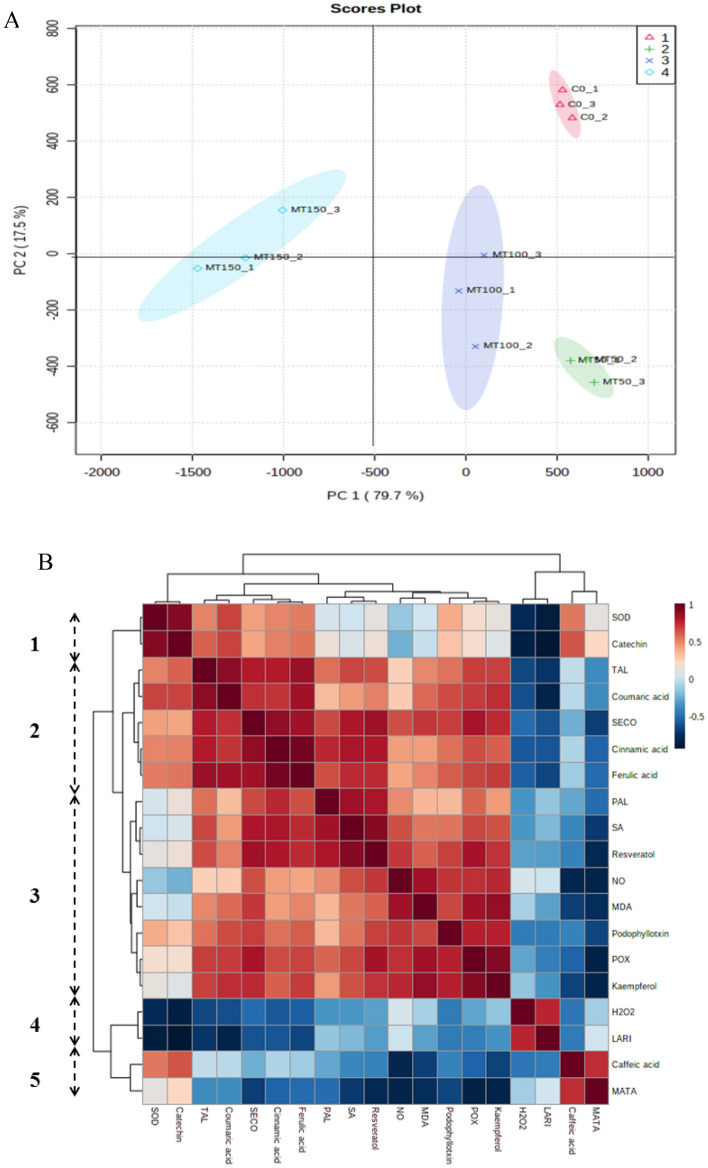
(**A**) Score (left) and loading (right) plots of the principal component analysis (PCA) conducted on the metabolomics data from samples treated with melatonin concentrations and the control. Each group of samples on the plot is indicated by an ellipse with 95% CI. (**B**) HCA map was employed for clustering of several metabolites and oxidative status according to Pearson correlations coefficient. Data are obtained from three replicates for each variation at all samples. Positive and negative correlations are described by red and blue colour, respectively. There are 5 basic clusters that are shown 1 to 5 on the picture.

For a better understanding the effects of melatonin on metabolic profiles, we analyzed the correlations amongst various metabolites through principal component analysis (PCA) (Fig. 5A). The loading plots provided by PCA showed that the metabolic profiles were successfully classified with an acceptable and excellent accuracy according to PCA results (the first component and second components were 79.7 and 17.5%, respectively). This analysis suggests clear separation based on samples at 50 and 100 µM of melatonin in the treated and untreated cells of *L*. *album*. The score graph (PC1 and PC2) of the PCA data emphasized that the separation of the sample outcomes was accomplished, and the variability of melatonin treatment (50 and 100 µM) is depicted by component 1. The response to melatonin treatment perspicuously had a significant percentage of the data set variability between different concentrations of melatonin treatment. Moreover, the metabolites provided from the tested cells were relegated on the basis of a similarity between the clusters pattern which was performed by the Pearson correlation coefficient (Fig. 5B). Enzymatic and non-enzymatic antioxidants were mostly relatively closely grouped, indicating that the observed distribution of samples is due to the concentrations of melatonin treatment (1–5). As the results, Fig. 5B explained that there are positive correlation patterns among various phenolic compounds in melatonin-treated *L*. *album* cells.

**Figure 6:**
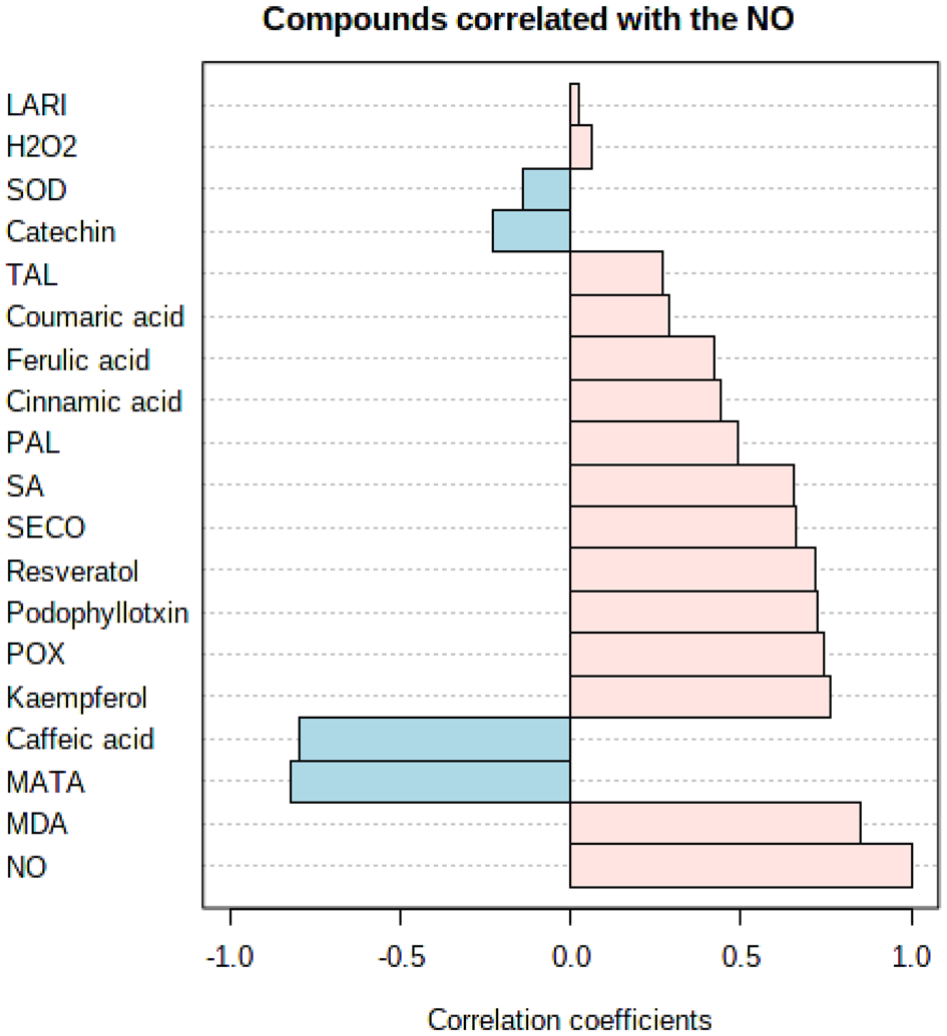
A summary of this research in which NO molecule can play a crucial role in melatonin responses in *L*. *album* cells. An increase in NO can affect H_2_O_2_ as a secondary messenger for inducing the phenylpropanoid pathway through enhancing SA production and the activity of PAL and TAL enzymes. Also, NO can provoke lipid peroxidation after melatonin treatment.

Finally, Fig. 6 is described a summary of this research, and we found that the NO molecule can play a crucial role in melatonin responses in *L*. *album* cells**.** An increase in NO can affect H_2_O_2_ as a secondary messenger for inducing the phenylpropanoid pathway by enhancing SA production and the activity of PAL enzyme. Also, NO can provoke lipid peroxidation after melatonin treatments.

## Discussion

Melatonin is a ubiquitous regulator molecule with multifunctional roles in the plant life processes, including tolerance against various environmental stresses^32^. Recently, there has been a large amount of literature describing that the exogenous application of melatonin can influence the plant defensive responses^4,5^. Despite enormous efforts to demonstrate the protective roles of melatonin in plant stress responses, the biological mechanisms of melatonin at the cellular level are not fully elucidated^33^. In this study, we illustrated that different concentrations of melatonin can induce enzymatic and non-enzymatic antioxidant reactions in* L*. *album* cells.

Defense mechanisms are triggered by many physiological processes, such as rising H_2_O_2_ content, increasing membrane permeability, releasing NO molecule, and producing secondary metabolites^13^. It is evident from the results that low concentrations of melatonin did not show significant effects on the content of H_2_O_2_, while it increased at the high dosage. These observations can be due to the inhibitory role of melatonin on the free radicals in* L*. *album* cells. This study confirms previous works on *Cucumis sativus*^34^ and *Salvia reuterana*^35^ that expressed melatonin may chiefly function as an essential regulator of ROS level, and detoxify excessive amounts of H_2_O_2_ molecule, suggesting that the low dosages of melatonin exhibiting no per-oxidative effects. In contrast, it has been reported that melatonin-induced H_2_O_2_ production can show a concentration-dependent behavior, and the high dosages of melatonin may presumably behave as a toxic element and reduce plant productivity^32,36^. This speculation is in agreement with our data indicating that H_2_O_2_ content can be enhanced through the high levels of melatonin in *L. album* cells.

On the other hand, melatonin treatment at low concentration (50 µM) noticeably accelerated the changes in NO generation *L. album* cells. The melatonin-induced NO generation in this study can be due to the up-regulation of nitrate reductase (NR) enzyme. In addition to converting nitrate to nitrite, this enzyme can reduce nitrite to NO using the NADPH cofactor^27^. Melatonin may indirectly modulate defense responses by leading a further increase in NO generation. Similar results were presented in *A. taliana* infected by the bacterial pathogen^37^, where the application of melatonin has a potential regulatory role in the NO generation, establishing signal transduction for the regulation of plant defense responses.

One more attribute appraised in the present study was MDA content which known as a lipid peroxidation indicator. Based on the observed resultants, melatonin-exposed *L*. *album* cells at 50 μM exhibited a significant elevation in MDA content as a dose-dependent response. At the same time, there were no significant differences in MDA levels at high concentrations of melatonin compared to the control. Therefore, from this point of view, we found that the highest content of NO under melatonin exposure caused an increased production of MDA in *L. album* cells. These results are in contrast to Soleimani Aghdam et al.^38^, who reported that interaction of melatonin with NO corresponded to the decreased MDA content, which may partially reflect in maintaining safe membrane integrity during chilling stress. In consistent with our findings, Kim et al.^39^ suggested that an increase in the endogenous NO level may promote oxidative damage, and thus raise the content of MDA.

Antioxidant enzymes play a key role in the plants defense machinery against various biotic and abiotic stress conditions. Melatonin noticeably improved the activity of antioxidant enzymes. Melatonin as a regulatory molecule can activate these enzymes and lead to balance of ROS levels and reduction of cellular oxidative damage^40^. In this experiment, the use of melatonin led to a change in the activity of antioxidant enzymes with different patterns in* L*. *album* cells. The activity of SOD enzyme as a source of H_2_O_2_ production, decreased in response to melatonin concentrations. In contrast, POD activity increased in the melatonin-treated *L*. *album* cells, which was coincided with the increased level of NO, representing that the activity of POD probably had a direct relationship with NO level. Furthermore, these changes in the activity of antioxidant enzymes can explain the changes trend in H_2_O_2_ level. An intriguing observation arising from the earlier studies^4,32,41^ is that melatonin positively motivates the activities of SOD and POD via the NO-dependent pathway. It has been suggested that melatonin can induce NO signaling pathway through interacting with unknown receptors, and subsequently regulate the function of various antioxidant enzymes and transcription factors^32^.

To get a better insight into the underlying mechanisms of melatonin in regulating the antioxidant agents, we performed a comparative metabolome analysis to characterize its functional role in inducing phenolic compounds in *L*. *album* cells. Likewise, PAL enzyme activity substantially increased in the presence of different concentrations of melatonin as a dose-dependent response. However, melatonin had no positive effect on TAL enzyme activity compared to the control group. The responses of PAL and TAL enzymes may differ due to their usage substrates. In addition, coumaric acid can be produced through an alternative pathway in which cinnamic acid is hydroxylated under the action of the cinnamate 4-hydroxylase (C4H) enzyme^42^. Over-activation of PAL enzyme resulted in a better supply of cinnamic acid for feeding into the phenylpropanoid pathway that was parallel with the increase of phenolic acids, flavonoids and lignans contents in the treated cells. It is important to mention that PAL and TAL are key enzyme to trigger the phenylpropanoid pathway via the provision of cinnamic acid and coumaric acid as primary precursors into other complex phenolics^10,23^. In accordance with our results, Duran et al.^43^ documented the significant effects of melatonin on PAL enzyme activity, promoting melatonin-induced defense reactions through the accumulation of phenolic compounds in *Ocimum basilicum*.

Interestingly, we found that content of defensive phytohormone SA increased in *L*. *album* cells in response to melatonin treatment. Melatonin can probably cause an increase the content of this phenolic hormone through regulating two key enzymes in the SA biosynthesis; PAL as a key enzyme in the production of phenolic compounds and also ICS-1 as the main and important enzyme in the biosynthesis of SA^27^. Overmeyer et al.^44^ also achieved that melatonin treatment triggers SA-induced defense signaling pathways by up-regulating SA biosynthesis genes such as isochorismate synthase-1 (ICS-1). On the other hand, SA enhancement can be a regulatory factor to adjust PAL activity and therefore, the modification of phenolic compounds production under the effect of melatonin could be a SA-dependent response^8^. Therefore, melatonin may be served as an up-regulator in the defense hormones signaling pathways, particularly SA. Their interaction likely led to strengthened defensive responses, indicating that the melatonin-mediated antioxidant activation in *L. album* cells not only limited to the NO molecule but may also derive from a complex interaction among melatonin, SA and NO signaling pathways. These results are consistent with Samari et al.^8^, who reported that biotic elicitor activates specific synergistic or antagonistic cross-talk amongst multiple signaling pathways, which trigger a complex network responsible for particular responses in various plant species. Our results can support the assumption that melatonin addition would positively affect phenolics accumulation through SA induction in *L*. *album* cells. As can be seen, there are many coincidences between both melatonin and SA molecules that their actions have aroused the production of phenolic metabolites, which are related to enhancing resistance to adverse situations.

In the current study, it was found that the profile of phenolic acids was robustly increased by melatonin treatment in *L*. *album* cells, but then declined with further increasing melatonin concentrations. The contents of cinnamic acid, coumaric acid, and ferulic acid reached their maximum at 50 and 100 μM melatonin treatments. The decrease in caffeic acid was since it may act as a prerequisite molecule in lignans biosynthesis. Also, our data showed a rapid increase in the contents of antioxidant compounds kaempferol and resveratrol in response to melatonin exposure, while the catechin level decreased. Several pieces of evidence in various literatures explained that exogenous melatonin treatment drastically induced secondary metabolites production derived from phenylalanine and tyrosine such as lignins, flavonoids, and lignans in different plant species^4,43^. Ultimately, we observed that melatonin can affect lignans accumulation differently in *L*. *album* cells. Based on our results, it appears that LARI is the lignan that is intensively accumulated in response to different concentrations of melatonin. In contrast, PTOX increased only at low concentration of melatonin, and the reduction of SECO and MATA contents as its precursor, in this concentration can be justified. The induction of lignans by elicitors has also been investigated in cell cultures of *L*. *album* in response to various elicitors^10,12,13,45^.

The HCA and PCA results further affirm that the melatonin can change the accumulation of phenolics, which peak at 50 and 100 µM in a dose-dependent manner. The PCA results revealed an overview of the differences between melatonin-treated and untreated cells. We observed a clear separation between the treatment parameters along the PC1, which consists 79.7% of the total variation. In accordance with these results, the increase in the contents of NO and SA, as well as the phenolic compounds, especially lignans under melatonin effect illustrates that the production of phenolic compounds can be significantly associated with the high levels of NO and SA. In this sense, it appears that *L*. *album* cells can respond to melatonin through the elevation of enzymatic and non-enzymatic defense mechanisms.

**Figure 7:**
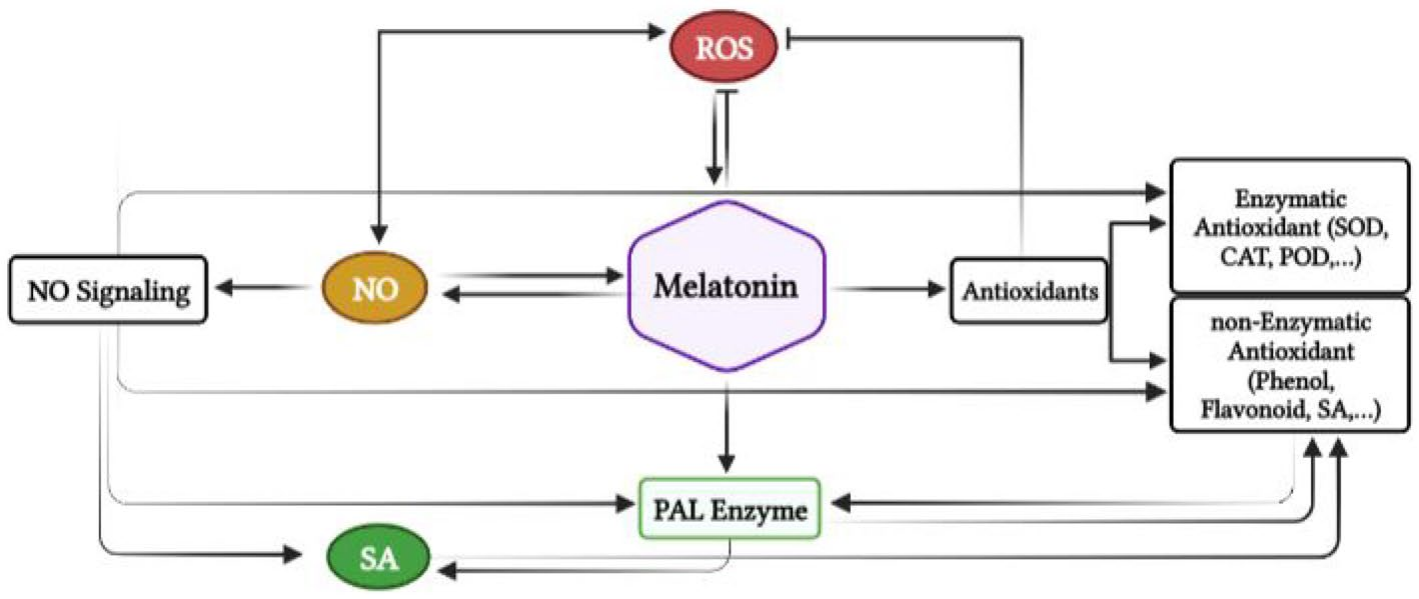
Schematic representation of a hypothetical model of melatonin-induced antioxidant machinery in *L. album* cell culture.

In summary, our observations confirm that melatonin can trigger complex network signaling pathways to strengthen antioxidant elements in *L*. *album* cells (Fig. 7). Based on our results, melatonin induces the NO and SA production as the regulatory molecules. It can be suggested that these regulatory agents in relationship to each other positively influences antioxidant enzymes activity and phenolic compounds production including phenolic acids, flavonoids and lignans in *L. album* treated cells. However, there is a need for more investigation to complete the attempt accomplished here to illustrate the detailed mechanism of melatonin in the regulation of defensive responses in this plant.

## Materials and methods

### Site description and collection of plant materials

All plant experiments were performed per the relevant institutional, national, and international guidelines and legislation. The plant materials were collected during June 2019 from wild-grown plants in the Sohanak region (35°480′N, 51°32′E, and altitude of 1900 m) placed in Tehran province, Iran. The permissions to harvest *L*. *album* plants in Tehran were provided. Besides they were discussed and approved by the Research Ethics Committee of Tarbiat Modares University. The formal identification of the *L*.* album* was done by Dr. Shahrokh Kazempour-Osaloo, a professor of plant systematic. A voucher specimen of *L*. *album* was kept at the Herbarium of department of Plant Biology of Tarbiat Modares University. The mature seeds were cultivated in MS medium for providing aseptic-plants as a source of explants.

### Cell culture and treatment

To provide* L*. *album* cell culture, sterile leaf-explant was applied as described in our previous report^46^. Briefly, MS basal medium^47^ fortified with 3% sucrose, NAA (2 mg L^−1^), and kinetin (0.4 mg L^−1^) solidified with agar (0.8%) was employed to culture the cells at 25 °C in the dark condition. The pH of all media was adjusted to 5.6 with NaOH (1 M) before autoclaving. Callus culture was regularly passage into fresh medium once a month. Suspension culture was prepared by transferring fragile calli into liquid MS medium (as mentioned above, but without agar, 30 mL in 100 mL Erlenmeyer flasks). All media were placed on a rotary shaker (100 rpm) at 25 °C in continuous dark condition. 2 g of cells were regularly sub-cultured every 8 days on the same medium based on a presentive growth curve (Data not shown).

Different concentrations of melatonin (0, 50, 100, and 150 µM) were utilized as treatment. Melatonin was dissolved in ethanol. After autoclaving, melatonin was added to the culture medium through filter-sterilized (2.2 µm) under aseptic condition (day of treatment). All samples were harvested after 5 days, and immediately chilled in –80 °C.

### Determination of H_2_O_2_, NO and, MDA contents

The accumulation of H_2_O_2_ was assayed according to Velikova et al.^48^ method. Briefly, 0.2 g of the fresh cells was extracted with 3 mL of 0.1% (w/v) trichloroacetic acid (TCA) on ice. Then, all samples were centrifuged at 12,000 rpm for 15 min, 4 °C. Subsequently, the supernatant (0.5 mL) was mixed with 0.5 mL of 100 mM phosphate buffer (pH, 7.0) and 1 mL of KI (0.1 M) in HCl (1 M) allowed standing for 1 h in the dark condition. The absorption was measured at 390 nm. The calculation of H_2_O_2_ content was done based on a standard curve, and the values were presented as μmol H_2_O_2_ g^−1^ FW.

The NO content was determined by measurement of nitrite concentration in vivo using Griess reagent^49^. 0.5 g of fresh cell was ground in 3 mL of 100 mM cool PO_4_^−3^ buffer (pH 7). The homogenates were centrifuged at 10,000 rpm for 15 min at 4 °C. Subsequently, the aliquot of samples (0.2 mL) was inoculated with 1.8 mL of PO_4_^−3^ buffer (pH 7) and 0.2 mL of Griess reagent (1% sulfanilamide and 0.1% N-1-naphthyl ethylenediamine dihydrochloride in 5% phosphoric acid solution) at 25 °C for 15 min. NO content was measured based on the absorbance of samples at 540 nm, and the concentration of NO was assessed by a sodium nitrite standard curve.

The formation of malondialdehyde (MDA), a lipid peroxidation marker, was performed based on Stewart and Bewley^48^ method with slight modificationx. In summary, 1 mL of cell extracts (in 0.1% TCA) and 0.5% thiobarbituric acid (in 20% TCA) were incubated for 30 min at 90 °C. Ultimately, the absorbance of mixtures was determined at 532 and 600 nm, and finally, the MDA accumulation was illustrated as μmol g^−1^ FW.

### Quantification of the activity of SOD and POD enzymes

Soluble proteins were quantified according to Bradford (1976) method^51^. SOD (EC 1.15.1.1) activity was assayed based on the inhibition of nitroblue tetazolium (NBT) reduction^52^. Also, POD activity (EC 1.11.1.7) was analyzed based on the quantity of guaiacol oxidation by H_2_O_2_ at 470 nm^53^.

### Measurement of PAL and TAL enzyme activities

PAL (4.3.1.5) and TAL (4.3.1.23) enzyme activities were analyzed using the method of Beaudoin-Eagan and Thorpe^54^ with some modifications. PAL activity was measured at 290 nm according to the formation of cinnamic acids, and TAL activity was assayed at 390 nm by the production of *p*-coumaric acid. The activity of PAL and TAL enzymes was estimated as µmol cinnamic acid and *p*-coumaric acid mg^−1^ protein min^−1^.

### Analysis of phenolic compounds by using HPLC

The compositions of phenolic acids were analyzed by Owen et al.^55^ method. At first, 0.2 g of fresh cell samples was extracted using methanol solvent in a crucible. After centrifuging at 12,000 rpm, the solvent of each sample was evaporated under ambient condition. The residue was solved in 4 mL acetonitrile. Subsequently, all samples were extracted three times with n-hexane (3 mL). The residue was resolved in 0.5 mL methanol to detect the phenolic acids through the HPLC system (Agilent Technologies 1260 infinity, USA). A C18 column (Perfectsil Target ODS-3 (5 μm), 250 × 4.6 mm; MZ Analysentechnik, Mainz, Germany) was applied for the stationary phase. The eluent system was comprised of 2% acetic acid in water (A) and methanol (B) with a gradient system, as explained by Tashackori et al.^56^. For phenolic acids quantification, a UV detector was set at 278 and 300 nm.

Individual flavonoids determination was performed based on Keinnen et al.^57^ method. Briefly, 0.2 g of fresh cell were ground to a fine powder, and then homogenized with 1.5 mL of 40% aqueous methanol supplemented with 0.5% acetic acid. These reaction mixtures were allowed to stand for 12 h, and then centrifuges at 13,000 rpm for 12 min. The supernatant was injected into HPLC system. In this analysis, the gradient mobile phase was composed of A: 0.5% phosphoric acid in deionized water and B: acetonitrile. To detect flavonoids, a UV detector was set at 254, 280, 300 and 350 nm^25^.

To evaluate lignans contents, 0.2 g of dried cell were extracted by methanol (80% v/v), as previously defined^46^. The extract was dissolved in 0.5 mL of methanol before HPLC analysis. The HPLC system was equipped with a C18-ODS3, 5 μm (250 × 4.6 mm) column. The elution solvent was acetonitrile and water with a gradient system following Ahmadian Chashmi et al.^58^.

### Statistical analysis

This experiment was done based on a completely randomized design with three replications independently. To analyze all data, SPSS 25 software was employed. Duncan's multiple range test was applied for determining statistical differences between means (*p* value ≤ 0.05). In addition, a data-matrix prepared which its rows represented samples and columns represented metabolites. To access more information about the metabolic profiling, the Principal Component Analysis (PCA) and Hierarchical Cluster Analysis (HCA) was computed through the algorithm embedded in the web-based software (http://www.metaboanalyst.ca).

## Data Availability

The data supporting the findings of this study are available from the corresponding authors, upon request.
